# On the Calabar Bean, by Christison

**Published:** 1864-05

**Authors:** 


### Selections.

### ON THE CALABAR BEAN, BY CHRISTISON; TH. R. FRASER; D. ARG. ROBERTSON; W. BOWMAN; J. W. OGLE; A. V. GRAEFE; E. HART; J. SOELBERG WELLS; J. HULKE; HARLEY. (From "Schmidt's Jahrbücher," Oct. 1863.)

Since a long while, the want of a remedy was felt, which acted antagonistically to atropine and the other mydriatics for producing a prompt contraction of the pupil. After various substances, particularly opium and ergotine, had proved ineffective, this remedy has been found in the Calabar Bean.

The drug was first mentioned by Robert Christison, in Edinburgh, in a paper "On the properties of the ordeal bean of old Calabar, West-Africa," published in 1855; but only its action, when taken internally, had then been examined. Th. R. Fraser, Assistant to the Professor of Materia Medica in Edinburgh, drew attention to the myotic qualities of the remedy. We copy from Fraser's remarks* the following:—

The bean comes from a *runner*, climbing on the bushes and trees, of the natural order—Leguminosæ; sub-order—Papilionaceæ; tribe—Euphasoleæ, growing in West-Africa, west of the river Niger (between 4° and 8° N. lat., and 6° and 12° E. long.), on the Calbary River. This region is inhabited by negro tribes. These call the bean Esere, and use it as an ordeal for criminals, in order to prove their innocence or guilt. The population of Calabar is estimated at 100,000, and of these upwards of 120 are reckoned annually to be thus sacrificed.

From the careful description of the plant (Physostigma venenosum, Balfour) I abstain, and merely mention that the root is spreading with numerous fibrils often having small succulent tubers attached. Inflorescence, axillary; on pendulous multifloral racemes; rachis of each raceme zig-zag and knotty. Calyx, campanulate, four cleft at apex, the upper division being notched and its segments ciliated. Corolla, papilionaceous; veined with a pale pink, having a purplish tinge, and curved in a crescentic manner. Stamens, ten, diadelphous. Pistil, more than one. Stigma, blunt, covered by a remarkable ventricular sac or hood, which extends along the upper part of the convexity of the style, having a resemblance to an "admiral's hat set in a jaunty manner." Legume, dark brown and straight, when mature, about seven inches in length, elliptico-oblong, with an apicular-curved point, and with outer and inner integument easily separable. Seeds two or three, separated from each other by a woolly substance.†

These so-called beans are on an average a little longer than one inch. They are irregular reniform, having the appearance of a somewhat flattened fusiform body bent on one of its edges. As obtained from Calabar, the beans have a gray color, and

* *Edinburgh Medical Journal*, VIII., IX., and X., 1863, a Monograph entitled "The Calabar Bean," Edinburgh, 1863.

† See Professor Balfour's paper in the Transactions of the Royal Society of Edinburgh, vol. XXII. Part II.

are incrusted with earthy matter. This is readily removed by washing, and a somewhat shining integument is exposed of various shades of brown, ranging from a light coffee to an almost perfect black.

The description of the kernel and spermoderm, consisting of three layers, I may dispense with.

While the other parts of the plant are indifferent, as it seems, to the animal organism, the beans have strongly poisonous qualities. According to the missionaries, those who have eaten them, first feel a violent thirst. Afterwards, the poisoned individual cannot swallow; mucus flows from the mouth; convulsions, particularly cramps in the muscles of the back, come on, etc. During all this, the patient is conscious of everything, and even the language remains up to shortly before death, which may ensue within half an hour. Sometimes vomiting occurs, after which the heat diminishes; and, except headache, all other symptoms disappear. Small doses (up to 12 grains) with which Christison made his experiments, soon caused an increasing pain in the epigastrium with retching, a sentiment of dyspnœa, cramps in the muscles of the breast, vertigo and weakness in the limbs, strong secretion of saliva, and irregular, slow motion of the heart, so that in one case the pulse made only 20 beats.

In rabbits, the alcoholic solution of the kernel of the bean, injected subcutaneously into the cellular tissue, produced, after five minutes, a copious discharge of urine. The fæces were eliminated normally in the beginning, but became more and more thin and watery; the hind legs were paralyzed, and the animal did not move when lifted up by the ears. After 20 minutes the pupils contracted, but not to the highest degree; they did also not cease to act somewhat on light. After 30 minutes, the animal would lie stretched out and respiration became noisy. During these experiments, the paralysis ceased after 2 to 3 hours, and only the diarrhœa and the strong secretion of urine remained for 12 hours.

Locally brought in contact with the muscles, the alcoholic extract produced loss of contractility; the intestines, painted over with a solution, ceased to move; in the cellular tissue it produced redness and inflammation, but not on the mucous membranes. Worms, which were also painted with the solution, first moved as if they had cramps and then died paralyzed; a partial paralysis could be produced by only painting them at isolated points. Internally the Calabar Bean (the extract prepared from the kernel) produced in the rabbit first slight shakings of the posterior extremities, then weakness thereof and evacuations of fæces and urine, contraction of the pupils, discharge of mucus from the mouth, and irregular respiration. Reflex movements cannot be produced. The animal finally seems dead, and the pupils dilate instantaneously. When it was opened, immediately the muscles of the extremities and the diaphragm were found to be yet contractable, and the heart also beat for 1 to 1½ hours longer; after this the left auricle ceased to move, then the ventricles, and finally the right auricle. The veins of the thorax were strongly injected; the lungs hyperæmic; in the stomach and intestines there was no change; liver and kidneys contained much blood; fauces and larynx were covered with mucus; there was much serum in the abdominal cavity; brain and spinal cord were unaltered, the membranes of the former hyperæmic.

Strong doses, given at once, caused instantaneous paralysis of the hind-legs, with slight cramps, flowing of mucus from the *nares*, strong secretion of tears, contraction of the pupils, and death after a few convulsive respirations. The pupils dilated immediately after death. In such cases there was no contractility of the muscles to be demonstrated in the cadaver; liver and lungs seemed normal; the intestines and bladder were filled; the heart in diastole, only contractable for a few moments by irritation; the peristaltic movements of the intestine were barely noticeable. In order to examine the local action of the remedy on the eye, Robertson* prepared an extract from the pulverized bean, which he dissolved in alcohol in three different concentrations. The weakest solution was produced by extracting 30 grains of the bean by alcohol, evaporating to desication and dissolving the rest in 1 dram of water. Thus a dirty light red-brown fluid was obtained. By further extracting and evaporating, a four times and an eight times stronger extract were obtained. After R. had examined his eyes and found that his pupils were 2 lines in diameter each, and that with each eye Jäger's No. I. was read at 5 inches distance, he put a drop of the weakest solution in his left eye, which did not produce any more irritation than a drop of water. After 10 minutes, objects at a distance of 1 foot became indistinct: at the same time all objects seemed larger and nearer. There existed also a sensation of tension in the eye, as if very minute objects had been assiduously looked at. Both pupils were yet equal in size. After 20 minutes, the left pupil had only a diameter of 1 line; objects further distant than 9 inches ap-

* *Edinburgh Medical Journal*, VIII., p. 815, March, 1863.

peared dim; every thing looked at seemed larger and nearer. The right eye was normal. After 30 minutes, the left pupil was only $\frac{2}{3}$, the right only $2\frac{1}{2}$ lines in diameter. The far-point of the left eye, 8 inches. After 50 minutes, the left pupil was $\frac{1}{2}$ and the right one 2 lines; a sensation of pressure and fatigue became manifest, when the subject of the experiment attempted to read; objects at 10 yards' distance were recognized with difficulty. After 6 hours, the left pupil was 1 line, the right one $1\frac{1}{2}$ lines; both reacted on light. After 12 hours, the left pupil was $1\frac{1}{2}$, the right one $2\frac{1}{2}$ lines in diameter. The following morning both pupils showed yet a slight difference, and the left eye was somewhat weak.

In a second experiment it was tried to find out the action of atropine when introduced into an eye, the pupil of which is at its maximum of contraction in consequence of the application of the extract of Calabar Bean. After contraction had been brought about in 25 minutes (there still existed some capacity of reaction), atropine produced dilatation to the normal size in about 2 to 3 hours. At the same time it was found that atropine in a solution of 2 grains to an ounce of water acts more strongly than the weakest solution of the extract of Calabar Bean, as the former in this experiment again dilated the pupil without a renewed application, after the pupil had been contracted anew by a second instillation of the solution of Calabar Bean. In a third experiment it was recognized that an already atropinized pupil begins to contract 10 minutes already after the application of the strongest solution of Calabar Bean, but that the action of the latter was not strong enough not to allow the action of the atropine again to appear; and that, on the contrary, a constant contraction of the pupil took place only after a second instillation of the extract of Calabar Bean had been made.

Bowman has also experimented on his own eye, as stated by Wells.* After 5 minutes, he felt a strong tension in the neighborhood of the ciliary body, as if something crept about there.† After 10 minutes this sensation had yet increased, and he felt also some lancinating pain. After 15 minutes, the near-point was, at the left side, $6\frac{3}{4}$ inches; while, in the right eye, to which nothing had been applied, it was removed to a distance of 15 inches. The far-point seemed equally distant in

* *Med. Times and Gazette*, May 16, 1863.

† Coetaneously with this sensation of creeping, I have frequently seen in patients twitching movements of single fibres of the musculus orbicularis palpebrarum.—[Ed.

both eyes. After 20 minutes, No. XVII. Jäger was seen at 15 feet, but the letters oscillated: they disappeared and returned alternately. The left pupil was then contracted to the size of the head of a pin, remained in this state for 18 hours; and, in the course of three days, again became normal. With this dilatation the reaction of the pupil on light became noticeable on both eyes. 25 minutes after the application, there existed astigmatism: the vertical staffs of a window appeared perfectly distinct at a distance of from 6 to 10 feet, while the horizontal ones seemed dim and angular. This was remedied by a concave cylindrical glass of 14 inches focus. With a cylindrical glass of 50 inches focus, distant objects appeared palpably smaller. This astigmatic state was yet found 18 hours after the application.

OGLE* has also made numerous experiments, which constantly showed the prompt contraction of the pupil after the application of the remedy. The action of the drug on the apparatus of accommodation, OGLE has not considered; and experiments in that direction are only then valuable if they are made upon sufficiently intelligent persons, or by physicians on themselves.

DE GRAEFE† has tested the new myotic on 9 healthy persons. The average time for the setting in of contraction was 14 minutes with the weak, 12 minutes with the strong solution; the duration of contraction with the former 2, with the latter 3 days; the maximum of contraction lasted from 6 to 18 hours. The altered state of refraction, *i. e.* the cramp of the muscle of accommodation and the approach of the near-point, lasted much less in GRAEFE'S experiments: it reached its height in 10 minutes, and remained there but from 10 to 20 minutes. The apparent increase in the size of objects and change of illumination were also observed; the acuteness of vision was reduced from 1 to $\frac{4}{5}$. Ophthalmoscopically there appeared no change of circulation. In a patient who had no iris, but good vision, the action on the ciliary muscle was also manifest. Experiments on birds showed the action of the drug on the pupil of these animals to be very brief; on amphibia and fishes the remedy remained without influence. DE GRAEFE also satisfied himself that atropine is a much more powerful irritant in an opposite sense than the Calabar Bean. The latter is not capable of contracting the pupil after it has just been dilated by atropine; the action of the latter also again appeared, when in an atropinized eye the Calabar Bean had for a short time

* *British Med. Journal*, June 13, 1863.

† Deutsche Klinik, 29. 1863.

produced a medium degree of contraction. When the pupil had first been contracted, atropine always acted, but somewhat slowly. The remedy acted also on the iris, when it was abnormal but not totally atrophic, f. i. in glaucoma and in a case of fistula of the cornea.

From all hitherto published experiments, it results that the Calabar Bean first produces a subjective sensation of tension in the ciliary body, which may be recognized also by the determination of the near-point and the range of accommodation; that it also causes contraction of the pupil; that the contraction reaches its height in the course of an hour; that the iris loses during that period its contractability; and that the dilatation to the normal size from this contracted state requires less time than the contraction of the pupil, when dilated by atropine (the latter circumstance probably depends on our incapacity up to the present time to extract entirely the active principles of the Bean). Simultaneously with the tension of the ciliary body occur the symptoms of myopia with a small range of accommodation, and of astigmatism. The remedy, therefore, acts by producing a cramp, by irritation of the ciliary branches of the oculo-motorius, in the ciliary muscle and sphincter of the pupil; it does not paralyze the dilatator pupillæ, as otherwise it could not produce complete contraction in a previously dilated pupil. It is, consequently, in so far an antagonist of atropine as the latter irritates the dilatator of the pupil.

Therapeutically the remedy will find the following applications: 1) Perhaps in retinitis, with great sensibility to light in order to moderate the admission of light. 2) In mydriasis, consecutive in some cases to debilitating diseases (Typhus, Diphtheritis, etc.) and to injuries. 3) In ulcers at the margin of the cornea, in order to avoid incarceration of the sphincter of the pupil after perforation. 4) In artificial mydriasis, in order to do away with the dazzling, which is very disagreeable to patients after having been examined by the ophthalmoscope, particularly if they have but one eye. [To these indications may be added the following: 5) In those corneal opacities with a transparent centre, which produce, when the pupil has its normal size, dazzling by diffusion of light. 6) In similar circumscript opacities of the crystalline body, situated near the centre of the latter, and in dislocations thereof. 7) In abnormal mobility of the lens, with a tendency to fall into the anterior chamber. 8) For the discovery of simulated amaurosis, the pupil being dilated with atropine. 9) In wounds of the cornea and sclerotic, with a recent prolapsus of the pheripheric

19

part of the iris. 10) Perhaps in diseases of the ciliary body.]

E. HART* has first made experiments in London with the Calabar Bean in paralytic mydriasis, and states that his experience entirely coincides with the facts already communicated by us. He considers it appropriate to instillate a fresh drop every 4 days; he himself used a solution of the extract of such a concentration that one drop contained the extractive matter of 3 grains of the crude drug. He furthermore says that the alcoholic extract is soluble in glycerine, and that this solution is more durable than the watery one, which already, after a few days, becomes decomposed.

WELLS had an opportunity to use the remedy in a woman of 26, who had rheumatic pain in the right half of the face; and, since three months, mydriasis on the same side, while the left eye was normal. We merely mention, from his very carefully communicated observations, that the affected pupil had a diameter of 4 lines and was immovable, and that the eye had a near-point of 18 inches for XVI. of Jäger's type. At the height of contraction, No. I. was seen from 10 to 12 inches, and a little afterwards from 8 to 10½ inches; the normal range of accommodation was therefore not re-established. When on the other (sound) eye the pupil was artificially contracted, it was determined that its near-point could be brought to 3 and its far-point to 5½ inches. From this it appears that the tension of the muscle of accommodation in the sound eye is artificially increased to a greater extent than in the eye affected with paresis of that muscle, as was to be expected. A cure of the disease in the patient was not effected, and it was necessary to apply every 3 or 4 days a fresh drop of extract of Calabar Bean.

HULKE has made experiments, as stated by HARLEY,† on three patients. In the one, who suffered from paralysis of both oculo-motor nerves in consequence of syphilitic periostitis of the orbit, the pupils contracted to 1 line, and the near-point approached from 20 to 12½ inches. In another, with mydriasis of the left eye of 4 years' standing, consecutive to a traumatic periostitis of the orbit, the pupil contracted from a diameter of 3 lines to ¾ of a line, and the near-point approached from 8½ to 6 inches. In the third case, with rheumatic paralysis of the oculomotor, the near-point was approached from 10 to 5 inches, and the pupil contracted to a diameter of 1 line.

—*Amer. Jour. of Ophthalmology.* GEISSLER.

* *Lancet,* No. 37, May 30, 1863.

† *Med. Times and Gazette,* June 30, 1863.

